# Delivery of monoclonal antibodies to the brain: the impact of nanocarrier structure

**DOI:** 10.1007/s13346-025-01957-y

**Published:** 2025-09-02

**Authors:** Laura Pineiro-Alonso, Inés Rubio-Prego, Ana M. López-Estévez, Pablo Garrido-Gil, Rita Valenzuela, José L. Labandeira-García, Pablo Aguiar, Ana I. Rodríguez-Pérez, María J. Alonso

**Affiliations:** 1 Center for Research in Molecular Medicine and Chronic Diseases (CiMUS), University of Santiago de Compostela, Health Research Institute of Santiago de Compostela (IDIS), Santiago de Compostela, Spain; 2 Department of Pharmacy and Pharmaceutical Technology, School of Pharmacy, University of Santiago de Compostela, Santiago de Compostela, Spain; 3 Networking Research Center of Neurodegenerative Diseases, CIBERNED, Madrid, Spain; 4 Instituto Madrileño de Estudios Avanzados en Nanociencia (IMDEA Nanociencia), Madrid, Spain

**Keywords:** Monoclonal antibodies, Nanomedicine, Brain delivery, Diffusivity

## Abstract

Monoclonal antibodies (mAbs) are promising therapeutic agents for neurological disorders due to their high specificity. However, their clinical application is significantly hindered by their poor transport across the blood-brain barrier (BBB) and their limited diffusion within the brain parenchyma. While significant efforts have been oriented to tackle the first barrier, the challenge of efficient brain diffusion remains largely underexplored. To address this, we have developed and evaluated two structurally distinct nanosystems for mAb delivery to the brain: PEGylated polyglutamic acid nanocapsules (PGA-PEG NCs) and PGAC14-based nanoassemblies (PGAC14 NAs). Both formulations encapsulated efficiently the model mAb bevacizumab (BVZ) while they exhibited different physicochemical properties. Namely, PGA-PEG NCs displayed a size of 80 nm and a neutral zeta potential, whereas PGAC14 NAs featured an ultra-small size of 40 nm and a negative surface charge. After assessing their diffusion capacity using immunofluorescence, we concluded that PGAC14 NAs exhibited the highest brain diffusion together with a favorable neuroinflammatory profile. This was likely driven by their small size and negative charge, along with a selective ability to interact with and deliver BVZ intracellularly to neuronal cells upon intraparenchymal administration. These findings provide key insights into optimizing nanocarrier design for improved mAb delivery to the brain.

**Supplementary Information:**

The online version contains supplementary material available at 10.1007/s13346-025-01957-y.

## Graphical Abstract

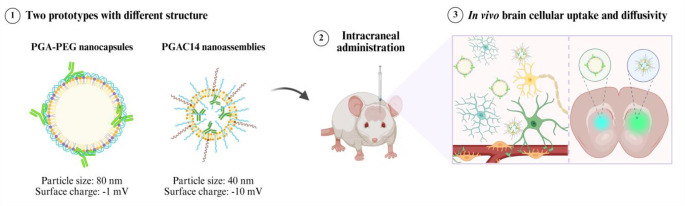

## Introduction

Monoclonal antibodies (mAbs) hold immense potential for treating neurological disorders, as evidenced by the recent FDA approval of three mAb-based therapies for Alzheimer disease and numerous ongoing clinical trials [1, 2]. However, their effective delivery to the brain faces substantial challenges [3, 4]. While much of the current research focuses on overcoming the blood-brain barrier (BBB), a critical obstacle for central nervous system (CNS) therapies, relies on the limited diffusivity of mAbs through the brain parenchyma. These barriers limit mAbs’ ability to reach their target sites within the brain, thereby reducing therapeutic efficacy.

To address these issues, nanotechnology has emerged as an enable technology for enhancing the delivery and distribution of therapeutic molecules within brain tissue [5–7]. While most studies have focused on the functionalization of nanocarriers for enhancing their transport across the BBB [8–11], the number studies addressing the diffusion of mAbs-associated nanocarriers across the brain tissue remains limited.

Pioneering work by Hanes, Suck and co-workers has shown that polymeric nanoparticles, particularly those incorporating polyethylene glycol (PEG), exhibit an efficient diffusion from the injection site into surrounding brain tissue [11–13]. This enhanced nanoparticle mobility within the brain parenchyma was attributed to their reduced opsonization and interactions with extracellular matrix components [14]. In this context, block copolymers composed of a biodegradable polypeptide blocks, such as poly(glutamic acid) (PGA) or poly(lysine) (PLL), combined with hydrophilic polymers, mostly PEG, have also emerged as promising polymeric therapeutics consisting of micellar formulations with various biomedical applications [15, 16]. Polypeptides, including PGA, possess advantageous properties such as biocompatibility, biodegradability, non-immunogenicity, and low toxicity, making them attractive candidates for drug delivery systems [17, 18]. Some examples of their potential are cisplatin-loaded PEG-PGA micelles, which have progressed to Phase III clinical trials for the treatment of advanced pancreatic cancer (NCT02043288) [19]. On the other hand, PGA-PEG nanocarriers have previously been explored for protein [20], and RNA delivery [17, 21].

Despite this previous work, to the best of our knowledge, this is the first report on the development of PGA-PEG-based nanosystems specifically designed for mAb delivery, particularly for brain-targeted applications. In the design of our PGA-PEG carrier, our hypothesis was that particle size and surface charge play a crucial role in the diffusion of nanosystems through the brain parenchyma’s extracellular matrix (ECM). This hypothesis relies on the reported information suggesting that to facilitate brain diffusion, the nanosystems should display a particle size below 100 nm, a dense PEG coating and a neutral or negative surface charge [22–24]. Moreover, although less studied, nanocarrier elasticity has also been suggested to influence diffusivity [8].

Based on this background information, one of the objectives of this work was to explore the utility of PGA-PEG nanocapsules (NCs) for the encapsulation and delivery of mAb to the brain. These NCs feature a hydrophilic PEGylated shell for prolonged stability and diffusivity, a vitamin E core to enhance deformability and brain parenchyma diffusion, and a positively charged phospholipid (DOTAP) to stabilize the structure and promote electrostatic interactions with the PGA shell. Using the model mAb bevacizumab (BVZ), we optimized the formulation to achieve high encapsulation efficiency, controlled release, and suitable physicochemical properties for CNS delivery.

On the other hand, building on previous work from our lab [25, 26], we explored a structurally distinct approach to mAb delivery. This approach was based on the assembling of PGA-C14 with mAbs and with the help of lecithin as a stabilizer. While both systems were engineered to encapsulate mAbs and facilitate brain delivery, their differences in structural composition offered a unique opportunity to investigate how nanosystem design influences brain diffusion and cellular uptake.

Through this comparative study, we aimed to sed light into the impact of nanosystem architecture and physicochemical properties on mAb brain diffusion and intracellular delivery, as well as to provide key insights into the design principles that could optimize nanosystem-based mAb therapies for CNS disorders.

## Materials and methods

### Materials

The humanized monoclonal antibody bevacizumab was kindly donated by mAbxience (Spain). Polyglutamic acid (PGA, nBuPGA(100)[Na]; molecular weight (Mp) determined by SEC-RI-MALS: 14.7 kDa; polydispersity index (Mw/Mn) by SEC-RI-MALS: 1.05) was provided by Curapath (Valencia, Spain). PGA-PEG (PEG (5 kDa)-b-PGA (10) [Na]) (polyethylene glycol (5 kDa)-block-poly(α-glumatic acid) (10u)sodium salt) was obtained from Polypeptide Therapeutic Solutions (Valencia, Spain). 1,2-dioleoyl-3-trimethylammonium-propane chloride (DOTAP) was obtained from Avanti Polar Lipids (Alabaster, AL, USA). Polysorbate 80 (Tween^®^ 80), vitamin E (D, L-α-tocopherol), Accutase^®^ solution, formaldehyde, penicillin/streptomycin solution, Amicon centrifuge filters (30–100 kDa), and glial fibrillary acidic protein (GFAP, 1:500, MAB360) were obtained from Merck (Darmstadt, Germany). Phosphatidylcholine from soybean (Lipoid S100) and N-(Carbonyl-methoxypolyethylene glycol-2000)-1,2-distearoyl-sn-glycero-3-phosphoethanolamine (DSPE-PEG2K) were purchased from Lipoid GmbH (Germany). Dulbecco’s Modified Eagle Medium (DMEM) and Opti-MEM were obtained from Gibco (Gaithersburg, MD, USA). Fetal bovine serum (FBS) and 24-well plates were purchased from Corning (New York, NY, USA). AlamarBlue Cell Viability Reagent and goat anti-human IgG FITC conjugate were obtained from Invitrogen (Massachusetts, MA, USA). NeuN antibody (clone A60, MAB360) and donkey serum, and Hoechst 33,342 (62249)were purchased from Sigma-Aldrich (San Luis, MO, USA). Ionized calcium-binding adaptor molecule 1 (Iba-1; ab178846) was obtained from Abcam (Cambridge, UK). Alexa Fluor 568-conjugated donkey anti-rabbit IgG or donkey anti-mouse IgG were purchased from Molecular Probes (Eugene, OR, USA). Oriole fluorescent gel stain was obtained from Bio-Rad (USA). Glial fibrillary acidic protein (GFAP, MAB360) was purchased from Merk Millipore (MA, USA).

GMP-grade zirconium-89 as [^89^Zr]Zr-oxalate in 1 M oxalic acid was purchased from PerkinElmer (BV Cyclotron, VU Amsterdam, Netherlands). Isothiocyanate derivative of Deferoxamine was obtained from CheMatech (Dijon, France). Oxalic acid (98%) and Immu-Mount were purchased from Thermo Fisher Scientific (Waltham, MA, USA). PD Imaging studies were conducted using a Bruker BioSpec 3T PET/MRI scanner (bore diameter: 17 cm) equipped with actively shielded gradients (450–900 mT/m). Centrifugal devices (300 kDa) were purchased from Pall Life Sciences (Port Washington, NY, USA).

### Preparation of BVZ-loaded nanosystems

#### Preparation of PGA-PEG NCs

NCs were formulated using a microfluidic approach (NanoAssemblr) in two steps. First, the organic phase, consisting of vitamin E (6.75 mg/mL), Tween^®^ 80 (1 mg/mL), and DOTAP (0.25 mg/mL), all dissolved in ethanol, was mixed with an aqueous phase containing BVZ (0.5 mg/mL in water). Subsequently, PGA-PEG (4 mg/mL) was added to the preformed formulation to complete the NCs formation. Finally, BVZ-loaded NPs were concentrated up to a final BVZ concentration of 1 mg/mL using a nitrogen source.

#### Preparation of PGAC14 NAs

Self-assemblies incorporating the PGAC14 amphiphilic polymer were prepared by adding 125 µL of an aqueous solution of BVZ (4 mg/mL) to 500 µL of an aqueous solution of PGAC14 (1 mg/mL) under magnetic stirring at 1100 rpm and RT. Subsequently, 50 µL of an ethanolic solution containing Lipoid S100 (20 mg/mL) and DSPE.PEG2K (2 mg/mL) in a ratio 1:1 v/v were added over the above mixture. Finally, BVZ-loaded NPs were concentrated up to a final BVZ concentration of 1 mg/mL using a nitrogen source.

### Physicochemical characterization of nanosystems

The physicochemical properties of all the prototypes were performed with a Malvern Zeta-Sizer (NanoZS, ZEN 3600, Malvern Instruments, Worcestershire, United Kingdom). The particle size and polydispersity index (PDI) were determined using Dynamic Light Scattering (DLS) after diluting accordingly the samples in H_2_O. The zeta potential was measured after dilution in PBS by Laser Doppler Anemometry (LDA), using the same device. For a further physicochemical characterization, PGA-PEG NCs were analyzed by Nanoparticle Tracking Analysis (NTA) after diluting the samples 10,000 times in H_2_O (NanoSight NS3000, Amesbury, United Kingdom).

### Quantification of the Mab association efficiency (AE) and loading capacity (LC) by using sodium dodecyl-sulfate polyacrylamide gel electrophoresis (SDS-PAGE)

The amount of associated mAb was determined by SDS-PAGE for separation under reducing conditions. PGA-PEG NCs were isolated by ultracentrifugation following the previously described conditions (i.e., 35000 rpm – 1.5 h – 15 °C). Then, the amount of non associated mAb present in the supernatant was quantified. Briefly, the supernatant was diluted accordingly to fit in the calibration curve. The mAb standard solutions of known concentrations (from 1 to 0.2 µg/lane) were prepared in PBS. Treated samples were diluted in a volume ratio 1:1 with 2X Laemmli buffer and vortexed for several seconds. Then, the samples were boiled at 99.5 °C for 5 min. 20 µL of the denatured samples were resolved using stacking and resolving polyacrylamide gels of 4 and 12%, respectively, in the presence of 0.1% SDS in the running buffer. After 1 h with variable milliamps and a voltage ranging from 100 to 120 V. Finally, samples were stained using Oriole fluorescent gel stain solution for 1 h at RT in head-to-tail rotation. Prior to analysis, the gel was washed twice with ultrapure water.

GelAnalyzer 19.1 software (www.gelanalyzer.com) by Istvan Lazar Jr., PhD and Istvan Lazar Sr., PhD, was used to process and analyze the samples. Total bands areas of the calibration curve were referred to the theoretical value and those ranging between 80 and 120% were considered. Samples’ bands intensities referred to the calibration curve. The AE and LC were determined as follows:

AE (%) = [1- (mass of free mAb) / theoretical mass of mAb] x 100.

LC (%) = [mass of associated mAb / total theoretical mass of the nanosystems] x 100.

### Dot blot for evaluation of antigen recognition

Several 4 µL droplets of Mili-Q water, recombinant antigen VEGF (0.05 µg) or the mAb BVZ (0.04 µg) were dispensed onto a pre-cut nitrocellulose membrane. Then, the membrane was blocked with blocking buffer (1× TBS with 5% w/v nonfat dry milk) for 1 h, washed three times with PBS/0.1% Tween 20, and incubated with free BVZ BVZ-loaded PGA-PEG NCs, BVZ-loaded PGA14 NAs or blank nanosystems for 1 h. Prior to the assay, the nanoparticles were disrupted to allow the release of the encapsulated antibody for antigen binding. After washing three times with PBS/0.1% Tween 20, the membrane was incubated with goat anti-human IgG FITC conjugate (1:500) for 1 h. Finally, the membrane was washed two times with TBS/0.1% Tween 20 and rinsed in PBS. Images were obtained by scanning the membrane with the ChemiDoc imaging system (Bio-Rad).

### Stability in simulated biological media

The colloidal stability of the PGA-PEG NCs and PGAC14 NAs was evaluated by diluting them 3-fold in PBS, followed by incubation at 37 °C under orbital shaking at 300 rpm. At specific time points (0, 2, 4, 8, and 24 h), the particle size and PDI were measured using DLS.

### AlexaFluor^488^ - BVZ conjugation

A total of 250 µL of BVZ (25 mg/mL) was mixed with AlexaFluor^488^ (10 mg/mL) in a molar ratio of 8:1 (AlexaFluor^488^: BVZ), using 1 M NaHCO₃ buffer (pH 8.5) to achieve a final volume of 300 µL. The reaction was mixed at RT for 5 min under low magnetic stirring, followed by overnight incubation at 4 °C. The conjugate was purified using Centripure MINI Desalt Z-50 columns (1000 g, 2 min) with 1x PBS, followed by concentration with 10 kDa MWCO centrifugal filters (14,000 g, 10 min, 15 °C) to a final volume of 100 µL. Samples were stored at 4 °C for short-term use or at -20 °C for long-term storage. The protein concentration (M) was determined by measuring absorbance (A) at 280 nm and 495 nm using a Beckman Coulter^®^ Du730 UV/VIS spectrophotometer, following the instructions from the supplier.

### DFO-BVZ conjugation

For radiolabeling the mAb, a first conjugation reaction was done between the mAb and the DFO [27]. Briefly, 1 mL of PBS containing 5 mg of mAb was mixed with 0.1 M Na_2_CO_3_ for an acceptable pH range of 8.9–9.1 and homogenized by vortex. Over this solution, 20 µL of 5 mM DFO dissolved in DMSO were added in 5µL steps. After 30 min at 37 °C, the final product was purified by 30–100 kDa centrifugal filters and washed with ultrapure water. Finally, the purified DFO-mAb conjugate was made up to 1 mL with ultrapure water.

### Radiolabeling with ^89^Zr

#### Radiolabeling of PGA-PEG NPs

A solution of ^89^Zr in oxalic acid 1 M was transferred into a metal-free tube, followed by the addition of Na_2_CO_3_ 2 M to increase the pH until 4–5. Then, an appropriate amount of HEPES 0.5 M (pH 7.4) buffer was added to neutralize the pH to 7 [28]. A solution of 150–200 µL of NCs was slightly added over the ^89^Zr solutions and the reaction mixture was kept under orbital stirring at 550 rpm for 1 h at 25 °C. The final product was purified using Amicon^®^ Ultra 0.5 mL Centrifugal Filters 100 K to remove free ^89^Zr.

#### Radiolabeling of PGAC14 NAs

The chelation of ^89^Zr to the DFO-BVZ was performed following a previously described method [27], which was adapted accordingly. 200 µL of 1 M oxalic acid containing ^89^Zr were neutralized with 90 µL of 2 M Na_2_CO_3_ pH 7–8 and incubated for 3 min at RT. Then, 500 µL of 0.5 M HEPES buffer pH 7.2, 700 µL of DFO-BVZ, and 500 µL of 0.5 M HEPES buffer pH 7.2 were consecutively added over the previous solution and mixed at RT under horizontal agitation at 350 rpm. After 1 h of incubation, the ^89^Zr radiolabeled-BVZ was concentrated using 30–100 kDa centrifugal filters to its original volume.

The ^89^Zr radiolabeled-BVZ entrapment into the nanoassemblies was performed as described in the section “Preparation of PGAC14 NAs”. Briefly, a non-radiolabeled BVZ was shifted by ^89^Zr radiolabeled-BVZ. Afterwards, the NAs were concentrated to a final mAb concentration of 1 mg/ by using a centrifugal device of 300 kDa (12000 g, 1.5 min, RT).

### Radiochemical characterization

After radiolabeling with ^89^Zr, the radiochemical yield (RCY%) was calculated as the percentage of radioactivity retained in the ^89^Zr-labeled nanosystems after purification compared to initial radioactivity used in the reaction (Figure S2A).

The percentage of radiochemical purity (RCP%) was assessed using instant thin-layer chromatography (iTLC) (Figure S2B). Briefly, 1.5 µl of ^89^Zr-labeled nanosystems was added in a glass microfiber chromatography paper impregnated with silica gel (instant TLC-SG Chromatography paper, Agilent, Santa Clara, CA, USA) and the paper was eluted with a solution of citric acid 20 mM + 60 mM EDTA: Acetonitrile (9:1 v/v). The radioactivity was measured by a TLC radioactivity detector system (RITA, Elysia-Raytest, Angleur, Belgium). Gina Star Software (RITA, Elysia-Raytest, Angleur, Belgium) was used to determine the ^89^Zr-labeled nanosystems which remained at the bottom of the paper and the free ^89^Zr, which is chelated by the EDTA and eluted with the eluents.

To evaluate radiochemical stability (RCS%), ^89^Zr-labeled nanosystems were incubated in PBS supplemented with 10% of FBS at 37 °C for 4, 24 and 48 h (Figure S2C). Following incubation, RCS% was determined as the percentage of radioactivity retained in the ^89^Zr-labeled nanosystems compared to free radioactivity after purification.

### Release profile of BVZ

The amount of released ^89^Zr-BVZ was quantified by gamma counter. The samples were radiolabeled with ^89^Zr and diluted 3 times in PBS at pH 7.4 and incubated under orbital agitation at 37 °C. Each time point was prepared in an independent low binding Eppendorf and at different time points, 500µL of the samples were centrifuged (10000 g, 15 min, 20 °C). The free antibody in the permeate and the BVZ-entrapped into the nanosystems were recovered and quantified by Gamma counter.

As control, free BVZ was treated in the same conditions and quantified. The release pattern was expressed as the % of cumulative BVZ released calculated as follows:

Cumulative BVZ released (%) = free BVZ at t1 - free BVZ at t0.

t0 indicates the condition before incubation, t1 corresponds to the condition at the time point.

under study.

### Cell viability assays

The toxicity profile of both prototypes was evaluated using the Resazurin assay (AlamarBlue Cell Viability Reagent). Primary monolayer cultures of astrocytes were kindly provided by Miguel López group [29]. Neurons (GT1-7 cells) and astrocytes were seeded in a 24-well plate at a density of 7 and 5 × 10^4^ cells per well and allowed to adhere overnight for 24 h at 37 °C with 5% CO_2_. Dulbecco’s Modified Eagle Medium (DMEM) (high glucose) containing 10% fetal bovine serum, and 5% penicillin/streptomycin was used as the cell culture medium. When the cells reached 70–80% confluence, the culture medium was removed and replaced with 400 µL of BVZ loaded nanosystems at various concentrations in DMEM. Cells co-cultured with nanosystems were incubated for 4 hours. Finally, Resazurin (1.22 mg/mL) was diluted in the cell culture medium to a final concentration of 12.2 µg/mL and added to each well. After 45 min of incubation, the fluorescence was measured using a microplate reader (Promega, Madison, WI, USA). Untreated cells served as positive controls, while cells treated with 0.5% (v/v) Triton X-100 diluted in cell culture medium were used as negative controls. The percentage of cell viability was calculated by subtracting the values of negative control from those of the samples and dividing by the fluorescence of the positive control.

$$\begin{aligned}&\mathrm{Cell}\:\mathrm{viability}\left(\%\right)\cr&=\frac{\mathrm{Fluorescence\:of\:the\:sample}}{\mathrm{Fluorescence\:of\:the\:positive\:control}}\end{aligned}$$

### Cell uptake

Neurons (GT1-7 cells) and primary cultures of astrocytes were seeded in a 24-well plate at densities of 7 and 5 × 10^4^ cells and allowed to adhere overnight for 24 h at 37 °C with 5% CO_2_. DMEM (high glucose) containing 10% fetal bovine serum, and 5% penicillin/streptomycin was used as the cell culture medium. When the cells reached 70–80% confluence, the culture medium was removed and replaced with 400 µL of BVZ loaded nanosystems at various concentrations in DMEM. Cells co-cultured with nanosystems were incubated for 4 hours. After 4 h of incubation, the cell supernatant was discarded, and cells were washed with cold PBS three times. Then, cells were detached from the wells with Accutase for 5 min at 37 °C and collected in FACS tubes. Thereafter, cells suspensions were washed with 3mL of cold FACS washing buffer (PBS 2% FBS) and centrifugated at 1000 rpm for 5 min. Finally, cells were fixed with 100µL of paraformaldehyde (PFA 4%) for 30 min. Treated cells were analyzed by flow cytometry in a FACScalibur instrument (10000 events) and resulting data were analyzed by FlowJo software BDLifescience.

### Animal studies

The animal study protocol was carried out following the European Communities Council Directive 2010/63/EU, Directive 86/609/EEC, and Spanish RD 526/2014, and was approved by the corresponding committee at the University of Santiago de Compostela (protocol 14,715,012/2021/012; last version 16 April 2021).

### Immunofluorescence study after intracranial administration

BVZ, PGA-PEG NCs, and PGAC14 NAs were administered intrastriatally to male Sprague-Dawley rats (8–10 weeks old, *n* = 6). Four animals were assigned to the PGA-PEG NCs/PGAC14 NAs group, receiving a PGA-PEG NCs injection in one striatum and a PGAC14 NAs injection in the contralateral striatum. Meanwhile, two animals were included in the control group, receiving BVZ in one striatum and PBS in the other. Before the surgical procedure, the animals were deeply anesthetized with ketamine (50 mg/kg) and medetomidine (0.4 mg/kg) and positioned in a stereotaxic frame (Kopf Instruments, CA, USA). A total volume of 2 µL of 1 mg/mL AlexaFluor^488^-BVZ, either encapsulated in PGA-PEG NCs, PGAC14 NAs, or in free form, was injected using a 10-µL Hamilton syringe attached to a motorized injector (Stoelting) at a rate of 0.5 µL/min. The stereotaxic coordinates used for the injections were A/P: 0.8 mm; M/L: 3.0 mm (right striatum) or -3.0 mm (left striatum) and D/V: 5.0 mm from dura. To minimize reflux, the needle was left in place for an additional 5 min before withdrawal. After 48 h, the animals were euthanized with an anesthetic overdose, followed by transcardial perfusion with 4% paraformaldehyde. Their brains were rapidly removed, cryoprotected, and cut into 30-µm thick coronal tissue sections using a cryostat. Brain tissue sections were collected as five series of adjacent sections, which were processed for histological analysis.

For diffusion quantification studies, one series of equally spaced (120 μm) sections covering the entire rostro caudal extent of the striatum were mounted on gelatin-coated slides and cover-slipped with Immu-Mount (Thermo-Shandon). Fluorescence images of each striatum were captured using a Nikon Optiphot-2 microscope with a 4x objective, a digital camera DXM1200 and ACT-1software.

For double immunofluorescence labeling, free-floating tissue sections were pre-incubated in KPBS-1% BSA containing 5% normal donkey serum and 0.03% Triton X-100 for 60 min at room temperature. Tissue sections were then incubated overnight at 4 °C with the corresponding primary antibody: NeuN antibody as a neuronal marker; GFAP, 1:500) as astrocytic marker; and Iba-1; 1:500) as microglial marker. The immunoreaction was visualized with the corresponding fluorescent secondary antibodies: Alexa Fluor 568-conjugated donkey anti-rabbit IgG (1:200) or donkey anti-mouse IgG (1:200). Finally, tissue sections were incubated for 30 min at RT with the DNA-binding dye Hoechst 33342 (1:2000) mounted on gelatin-coated slides, coverslipped with Immu-Mount and were visualized with a confocal laser-scanning microscope (AOBS-SP5X; Leica Microsystems Heidelberg GmbH, Mannheim, Germany).

### Quantification of fluorescence images

For diffusion quantification studies, a series of equally spaced (120 μm) sections covering the entire rostrocaudal extent of the striatum (30 μm thickness) were analyzed. The fluorescence area in each consecutive brain slice was precisely outlined and quantified using ImageJ. The total diffusion volume was then calculated using the following formula:

$$\begin{aligned}&Volume\:of\:distribution\:\left({\mu\:m}^{3}\right)\cr &=\mathop \sum \limits^{n}Area\:of\:fluorescence\:\left({\mu\:m}^{2}\right)\cr &*150\left(\mu\:m\right)*n\:\:\end{aligned}$$

### Assessment of inflammation in peri-injection regions

To quantitatively assess astrocytic and microglial responses in peri-injection areas, we conducted an immunofluorescence analysis combined with standardized image acquisition and semi-automated quantification. Coronal brain images processed for immunofluorescence for GFAP and Iba1 were acquired using a Nikon optiphot-2 microscope equipped with a Nikon Digital camera DXM 1200. For astrocyte analysis, images were taken at 4× magnification; for microglia, 10× magnification was used. Per animal, a minimum of five non-overlapping fields were selected from anatomically matched peri-injection regions.

Quantification was performed using ImageJ software (NIH, Bethesda, MD, USA). Images were converted to 8-bit grayscale, and a consistent threshold was applied to isolate immunopositive signal. Cell counts were obtained semi-automatically using the ‘Analyze Particles’ function, with defined size and circularity parameters to exclude artifacts and background noise. Data are presented as the number of immunopositive cells per square millimeter (cells/mm²). All image acquisition and analyses were performed by investigators blinded to the experimental groups.

### Statistical analysis

All statistical analyses were conducted using GraphPad Prim version 9.3.0. Gaussian distribution was determined using a Shapiro-Wilk test. The differences were considered significant for * *p* < 0.05, ** *p* < 0.01, *** *p* < 0.001, and **** *p* < 0.0001 with a confidence level of 0.05. The statistical analysis details are provided in the corresponding figure legends.

## Results and discussion

To assess the impact of the composition and physicochemical properties of the selected nanocarriers, PEG-PGA NCs and PGAC14 NAs, on their diffusion capacity, we conducted a comparative analysis of their physicochemical properties, drug loading and cellular uptake in vitro, and in vivo behavior, including cellular interactions and brain diffusion.

### Development and physicochemical characterization of PGA-PEG nanocapsules

Building on our lab’s expertise in NCs development [30–33], we designed NCs tailored for the encapsulation and delivery of mAbs to the CNS, with an emphasis on promoting widespread brain tissue distribution and effective interaction with brain cells.

**Fig. 1:**
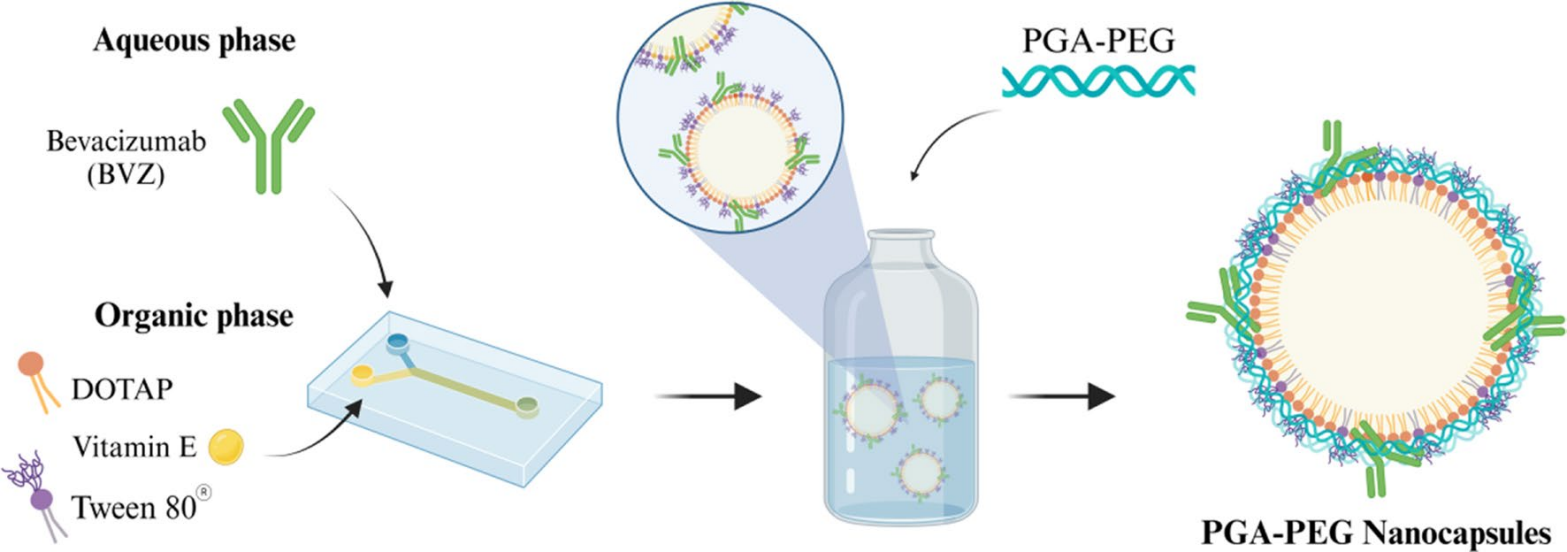
Schematic representation of the formulation method carried out for the development of PGA-PEG NCs

Previous studies have shown that PGA-based nanoconjugates bearing propargylamine moieties and bisdemethoxycurcumin not only were able to cross the BBB but also diffuse through the brain parenchyma and ultimately being internalized by brain cells [34, 35]. These findings, along with the exceptional properties of polypeptides [17], motivated us to develop PGA-based nanocarriers for the efficient encapsulation of the mAb BVZ. In particular, we developed PGA-PEG NCs with a vitamin E core surrounded by a PGA-PEG shell (Fig. 1). The hypothesis was that the PEGylated shell in combination with Tween^®^ 80 would enhance stability and favor the diffusion across the brain tissue [36]. Additionally, DOTAP was incorporated in the oily core to facilitate the electrostatic attachment of PGA-PEG to the NC structure [37], while vitamin E was expected to confer the NCs with an adequate deformability [8].

Hence, different combinations of PGA-PEG, DOTAP, vitamin E and Tween^®^ 80 were initially screened leading to the identification of a final prototype with a particle size of 98 nm, PDI < 0.2 and a ζ-Potential of -6 mV (Table 1). Given that particle sizes below 100 nm have been described to ensure adequate distribution within the brain parenchyma [3, 4], we explored microfluidic technology as a strategy to reduce the particle size of our final prototype.

**Table 1:** Physicochemical properties, association efficiency (AE%) and loading capacity (LC %) of PGA-PEG NCs after at a final BVZ concentration of 1 mg/ml. Data are expressed as mean ± sd, *n* ≥ 3. Statistical analysis was evaluated by using an unpaired t-test

Prototype	Final composition				Physicochemical properties			AE (%)	LC (%)
	PGA-PEG	DOTAP	Vitamin E	Tween 80^®^	Particle size (nm)	PDI	ζ potential (mV)		
PGA-PEG NC	4	0.25	6.75	1	93 ± 6	0.10	-1 ± 1	44 ± 12	2
PGA-PEG NC microfluidics					78 ± 3	0.06	-1 ± 1	99 ± 14**	4

As shown in Table 1, the use of microfluidics resulted in a reduction in particle size, obtaining NCs of 78 nm. Notably, the most significant optimization was observed in the AE%, which exhibited a marked increase, exceeding 90%. This improvement could be a result of the precise, continuous, and rapid mixing facilitated by microfluidics, promoting uniform formulation and improving nanoparticle self-assembly while minimizing compound loss [38, 39]. In consequence, the LC% was doubled, highlighting the efficacy of microfluidics in optimizing nanoparticle performance.

**Fig. 2:**
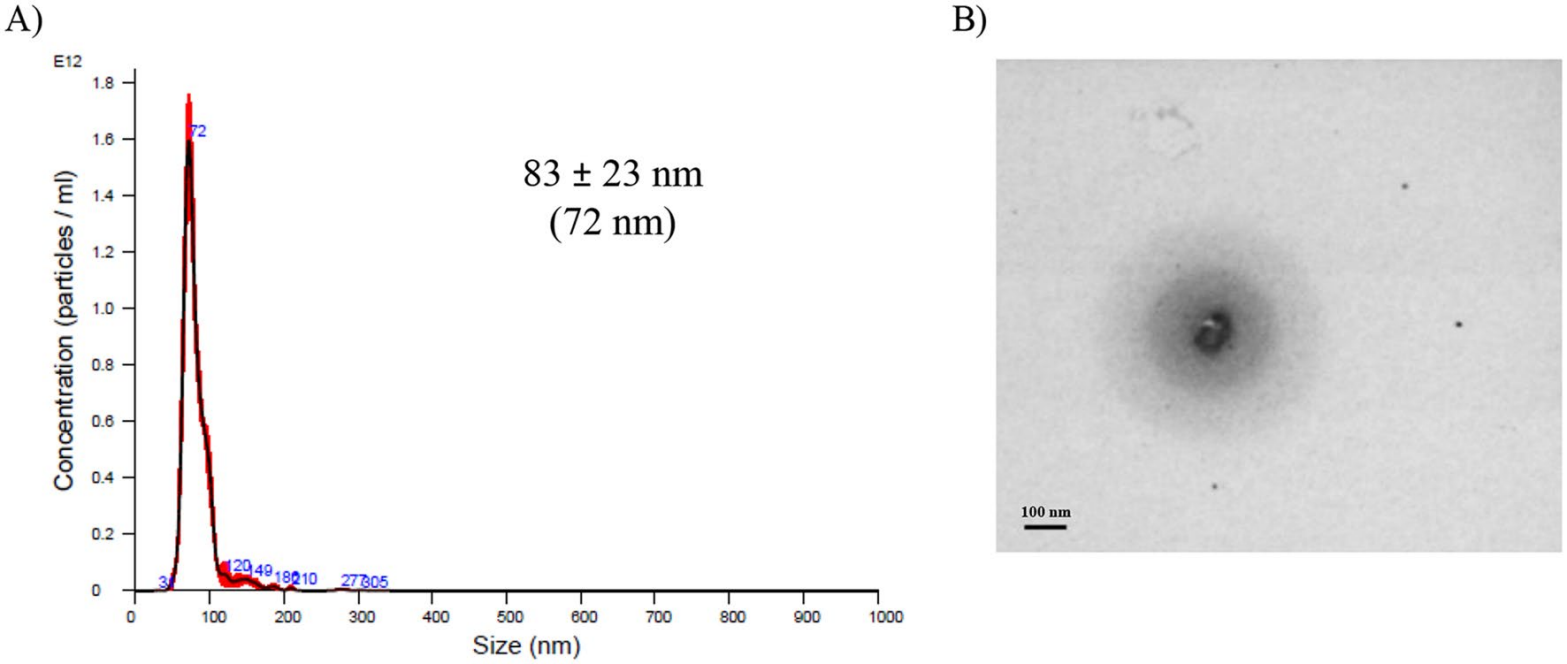
Graphs size distribution of BVZ-loaded PGA-PEG NCs by NTA (**A**) and STEM (**B**). Data is expressed as mean ± SD (mode), *n* ≥ 2. Scale bar 200 nm

An additional characterization of the particle size of PGA-PEG NCs was conducted using NTA. The results in Fig. 2A show their consistency with those obtained by DLS, confirming the presence of a monodisperse and stable (high dilution) NP population.

In addition, PGA-PEG NCs were analyzed by scanning transmission electron microscopy (STEM), revealing a spherical morphology with a distinct dark core surrounded by a lighter shell, likely composed of PGA-PEG (Fig. 2B). Particle size analysis showed a larger size, 117 ± 37 nm, compared to NTA/DLS measurements. This discrepancy may be attributed to sample preparation procedures, as drying and staining processes can influence nanoparticle characterization.

### Development and physicochemical characterization of PGAC14 nanoassemblies

**Fig. 3:**
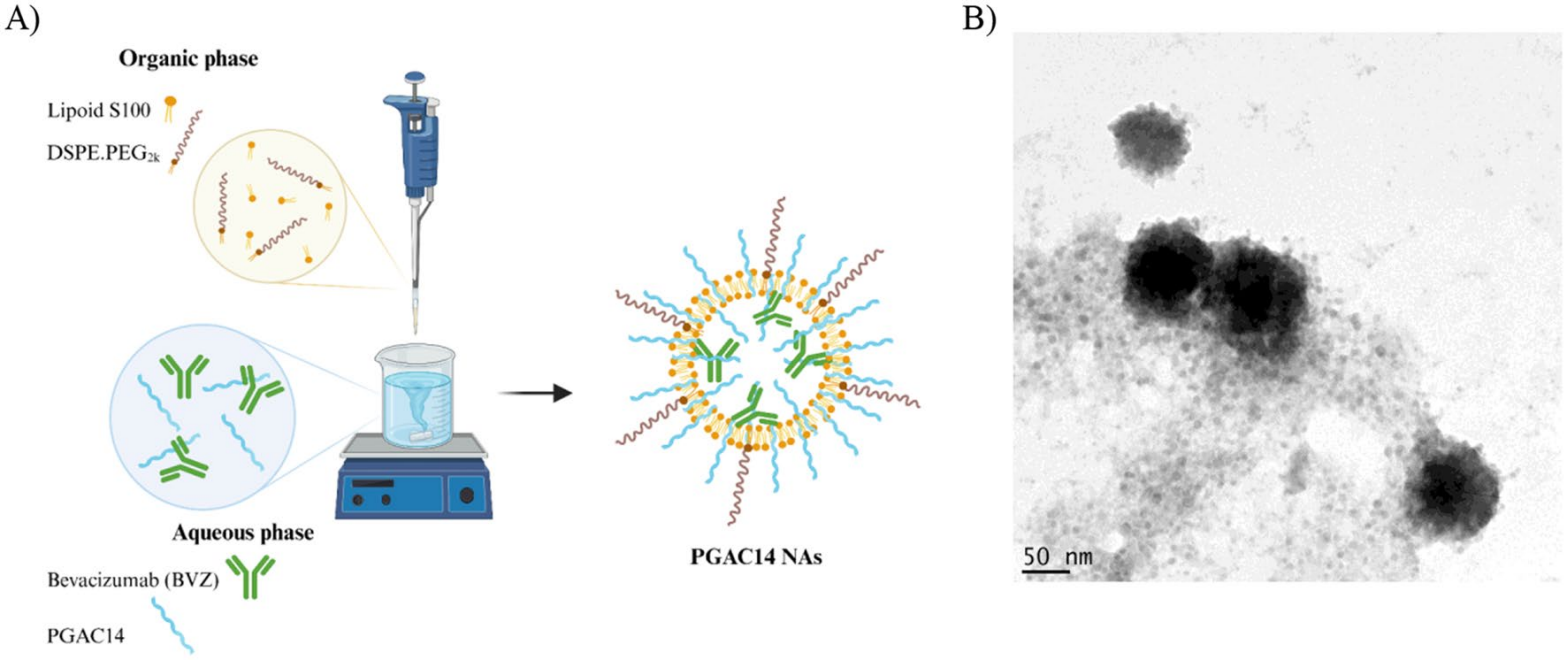
Schematic representation (**A**) and representative TEM images (**B**) of BVZ-loaded PGAC14 NAs. Scale bar: 50 nm

To evaluate how the nanosystem’s structure affects diffusion through the brain parenchyma, as well as its interaction with brain cells and intracellular mAb delivery, we selected a second prototype, PGAC14 NAs, for comparison (Fig. 3). These nanosystems have been previously developed in our group for the oral delivery of mAbs (A.M López-Estévez et al., In press).

The primary reason for selecting this nanosystem was its similarity in terms of surface composition to PGA-PEG NCs, as both incorporate the PGA polymer as the key component driving mAb interaction and assembly. Additionally, PGAC14 NAs contained PEG, which, as previously mentioned, enhance nanoparticle diffusion through the brain parenchyma by reducing interactions with extracellular matrix components [14].

The main difference between these systems lies in their core composition and physicochemical properties. As shown in Fig. 3, PGAC14 NAs possessed a hydrophilic core enclosed by a bilayer, with the mAb playing a crucial role in assembly and conformation, while PGA-PEG NCs featured a vitamin E core surrounded by a PGA-PEG polymer shell, as described in the section “Development and physicochemical characterization of PGA-PEG nanocapsules”. Additionally, PGAC14 NAs exhibit ultra-small particle sizes, homogeneous populations, and a slightly negative surface charge (Table 2).

**Table 2:** Physicochemical properties, association efficiency (AE%) and loading capacity (LC %) of PGA-PEG NCs AND PGAC14 nas. Data are expressed as mean ± sd, *n* ≥ 3. Statistical analysis was evaluated by using an unpaired t-test for comparison of particle size, PDI and ζ potential; and Mann-Whitney test for comparison of AE% and LC%

Prototype	Physicochemical properties			AE (%)
	Particle size (nm)	PDI	ζ potential (mV)	
PGA-PEG NCs	78 ± 3	0.06	-1 ± 1	99 ± 14
PGAC14 NAs	44^****^ ± 4	0.24^**^	-10 ± 2	61^*^ ± 9

Regarding the AE%, PGAC14 NAs exhibited a lower encapsulation efficiency compared to PGA-PEG NCs. However, their LC reached 20% (w/w), an outstanding value compared to other protein-delivery nanocarriers, which typically do not exceed an LC of 10% [40–42].

Notably, the BVZ encapsulated in both nanosystems remained functionally active upon encapsulation, as evidenced by its preserved ability to recognize and bind to its target antigen (Figure S1). These results indicate that the formulation process did not compromise the structural or functional integrity of the antibody.

Altogether, these features enabled a direct comparison of how structural and physicochemical differences influence brain diffusion and cellular uptake, further guiding the optimization of nanocarrier-based strategies for mAb delivery.

### Colloidal stability in simulated biological medium and release profile in vitro

To better understand the in vivo behavior of PGA-PEG NCs and PGAC14 NAs following intracranial administration, as well as the BVZ release profile, both nanosystems were incubated in PBS at 37 °C to simulate physiological conditions, given that these nanocarriers will be directly administered into the striatum.

**Fig. 4:**
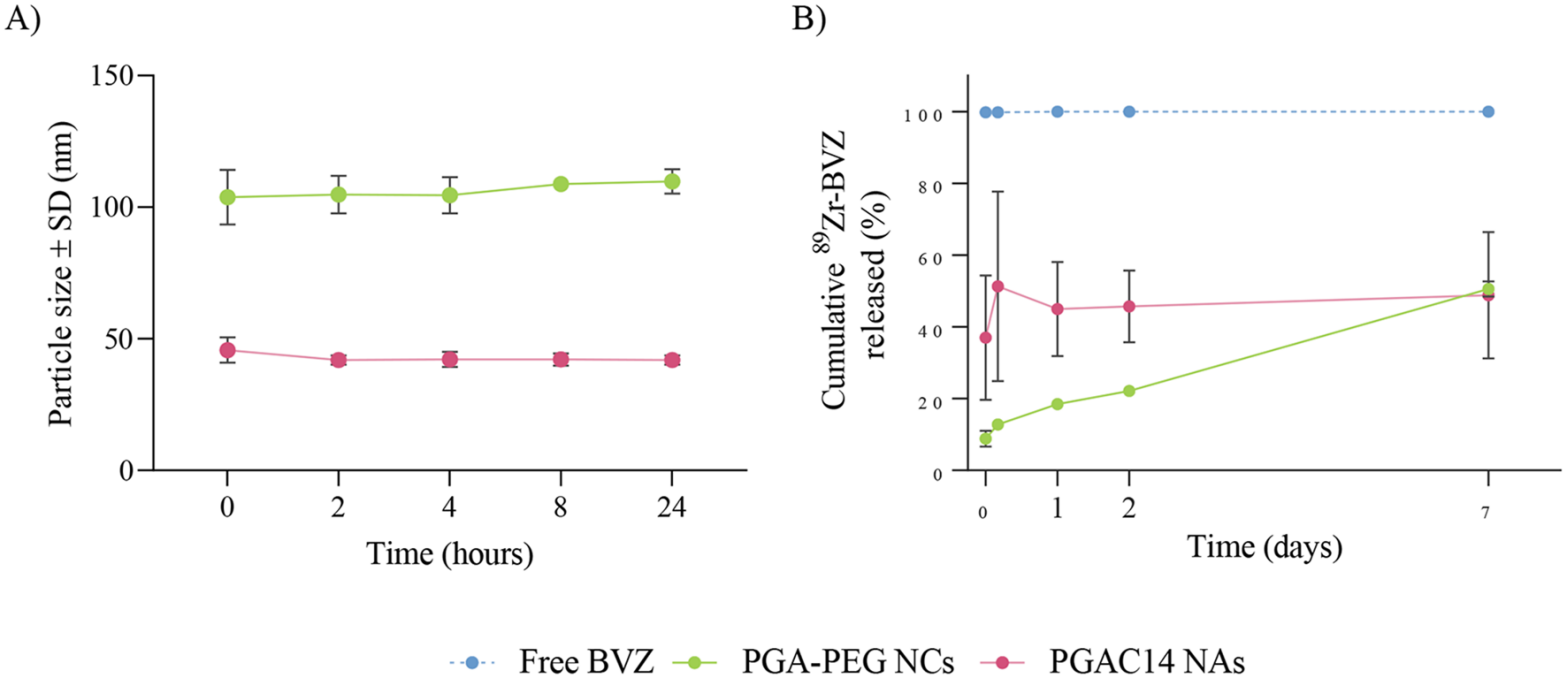
Colloidal stability of PGA-PEG NCs and PGA NA and BVZ release overtime upon incubation in PBS at 37 °C. **A**) Colloidal stability of both nanosystems was monitored overtime by DLS. **B**) The cumulative amount of released ^89^Zr-labeled BVZ was quantified overtime by Gamma counter. As a control, free BVZ was treated in the same conditions. Data is represented as mean ± SEM, *n* ≥ 2. Statistical analysis was done using a multiple unpaired t-test for comparison of PGA-PEG NCs vs. PGAC14 NAs and multiple Mann-Whitney test for comparison of PGA-PEG NCs or PGAC14 NAs vs. free BVZ

First, their colloidal stability was evaluated. As shown in Fig. 4A, both nanosystems remained stable for up to 24 h. PGA-PEG NCs exhibited a slight initial increase in particle size before stabilizing, whereas PGAC14 NAs showed no significant alterations throughout the incubation period.

Beyond stability, the ability to efficiently encapsulate BVZ and enable controlled release in biological fluids is a critical advantage of nanocarrier-based drug delivery [43, 44]. As shown in Fig. 4B, BVZ release from PGA-PEG NCs followed a controlled and gradual pattern, with an initial release of 9 ± 4%, progressively increasing to 51 ± 4% over 1 week. This sustained release profile minimizes burst release, potentially reducing the rapid clearance of free BVZ from the brain and enhancing intracellular delivery to target cells distant from the injection site.

In contrast, PGAC14 NAs exhibited a rapid initial burst release of 37 ± 30%, which then plateaued with minimal additional release up to 1 week, ultimately reaching 49 ± 31%. Despite similar final release values, these results indicate that PGA-PEG NCs are able to better control the release of BVZ overtime. However, these findings should be interpreted with caution, as in vitro results do not always correlate with in vivo behavior. In vitro conditions provide an initial understanding of how effectively BVZ is entrapped within the nanosystem and its release over time, but they cannot be considered as definitive predictions for in vivo performance.

### Impact of nanosystems on brain cell viability and mAb internalization

Assessing the impact of nanosystems on brain cell viability and their ability to facilitate mAb internalization is important for determining their therapeutic potential. While efficient cellular uptake is essential for intracellular drug delivery, ensuring minimal cytotoxicity is equally important. In this study, both prototypes were prepared with AlexaFluor^488^-conjugated BVZ (Table S1) to enable this in vitro evaluation in neurons and astrocytes.

**Fig. 5:**
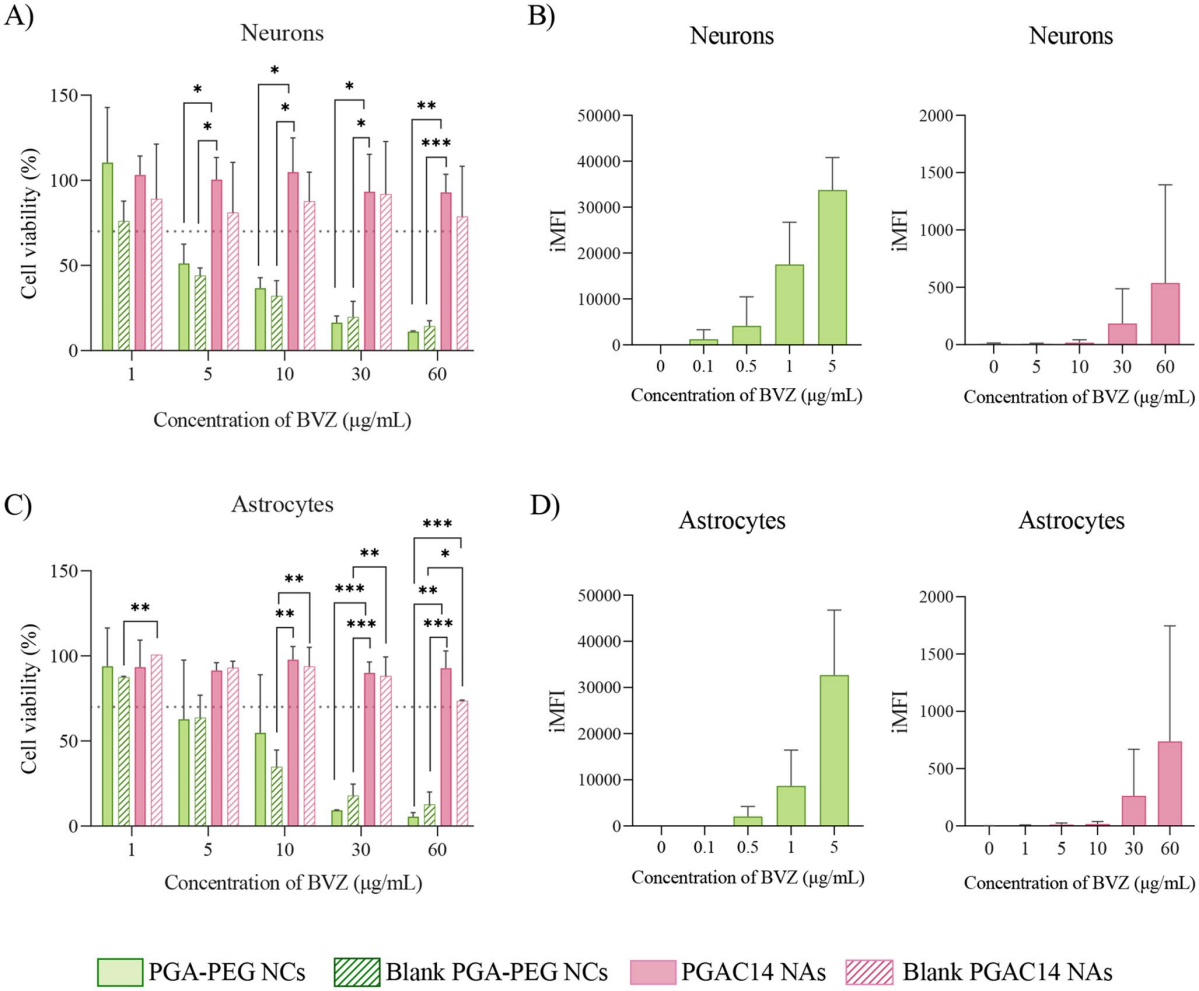
Metabolic activity and cellular uptake of PGA-PEG NCs and PGAC14 NAs loaded with AlexaFluor^488^-labeled BVZ in neurons (**A**-**B**) and astrocytes (**C**-**D**) after 4 h of exposure. Cells were exposed to increasing concentrations of blank and BVZ-loaded nanosystems. Cell viability values below 70% were considered toxic (dashed lines). Data are expressed as mean ± SD, *n* ≥ 3. Statistical analysis for comparison between groups was done using a unpaired t-test (A and C). iMFI integrated mean fluorescence intensity

To evaluate cytotoxicity, a resazurin reduction assay was performed to assess metabolic activity 4 h post-treatment in neurons (Fig. 5A) and astrocytes (Fig. 5C). Cells were exposed to increasing BVZ concentrations (1–60 µg/mL), with a 30% reduction in cell viability considered toxic (ISO 10993-5 standards) [45].

As shown in Fig. 5, both nanosystems exhibited distinct toxicity profiles across the tested cell types at equivalent BVZ concentrations. PGAC14 NAs demonstrated consistently low toxicity across all tested doses whereas PGA-PEG NCs exhibited a dose-dependent decrease in cell viability, remaining above 70% at BVZ concentrations ≤ 1 µg/mL in both cell types. This decrease is likely driven by the composition of PGA-PEG NCs, particularly the presence of cationic lipids, which has been associated with increased cytotoxicity at high concentrations [46].

However, cytotoxicity should be considered alongside cellular uptake, which was significantly higher in PGA-PEG NCs compared to PGAC14 NAs (Fig. 5B and D). The high uptake of PGA-PEG NCs, even at low BVZ doses (0.5 µg/mL), may have contributed to increased cytotoxicity, potentially via elevated reactive oxygen species (ROS) production [47]. In contrast, PGAC14 NAs required a BVZ dose 120 times greater to achieve similar integrated mean fluorescence intensity (iMFI) levels as PGA-PEG NCs.

The enhanced BVZ internalization observed for PGA-PEG NCs may be attributed to the composition of these NCs, which include compounds such as Tween^®^ 80, known to facilitate cellular uptake [48, 49]. Additionally, while particle size is often cited as a key factor in cellular uptake [50, 51], our findings do not support a direct correlation, as PGAC14 NAs, despite their smaller size, exhibited lower internalization. Another important property that might contribute to their uptake is their elastic behavior. Notably, PGAC14 NAs maintained high cell viability even at elevated doses, suggesting that increasing their treatment concentration could enhance BVZ intracellular delivery without compromising cell viability in vitro.

### In vivo assessment of mAb-loaded nanosystems diffusion in the brain by fluorescence

**Fig. 6:**
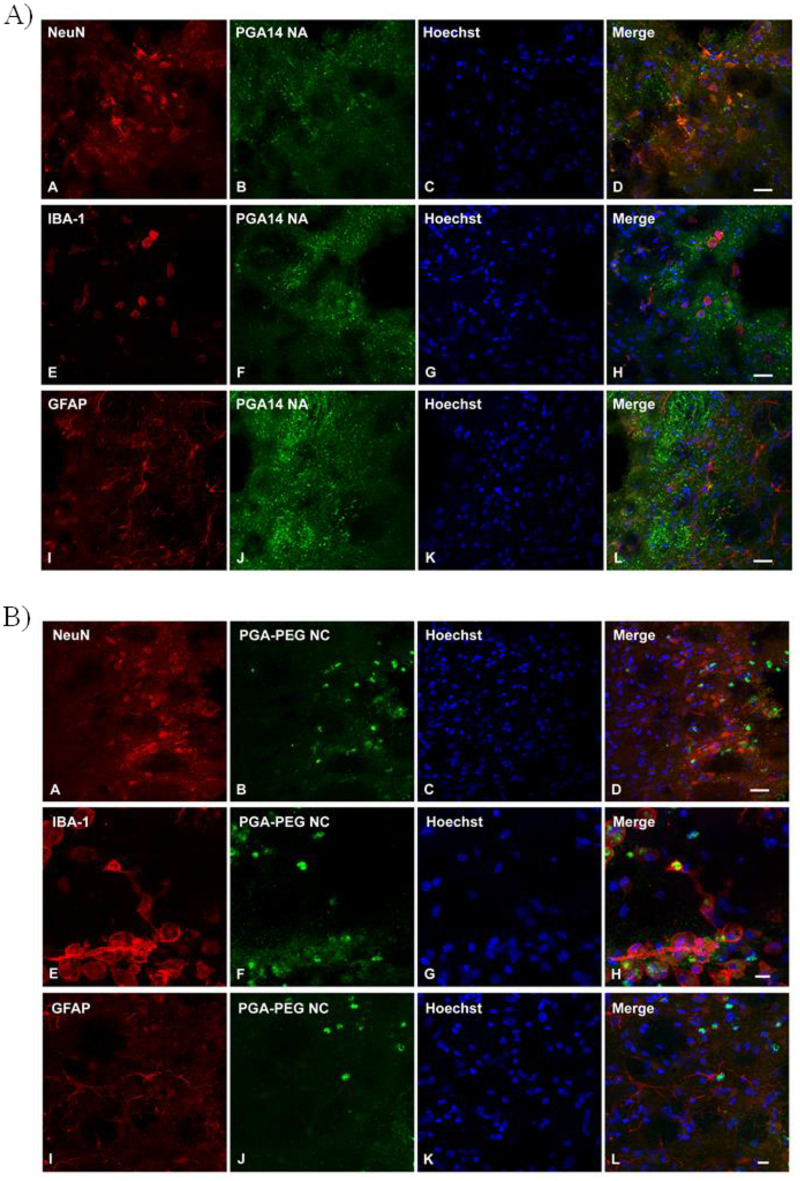
Internalization of NPs in neuronal and glial cells in brain tissue following administration of PGA14 NAs (**A**) or PGA-PEG NCs (**B**). Triple immunofluorescent labeling for different cellular markers in red (NeuN as neuronal marker [A]; IBA-1 as microglial marker [E] and GFAP as astroglial marker [I]), the nuclear counterstaining Hoechst (in blue; [**C**, **G**, **K**]), and AlexaFluor^488^-labeled PGA14 NAs (top panel; [**B**, **F**, **J**]) or PGA-PEG NCs (bottom panel [**B**, **F**, **J**] (in green). Merged images (**D**, **H**, **L**) demonstrate co-localization of nanoparticles with various cell populations, indicating intracellular uptake. Scale bar: 20 μm

The objective of this study was to compare the efficiency of two nanosystems with distinct nanostructures but sharing some similar components, in terms of their capacity to diffuse across the brain and deliver the model mAb BVZ inside the brain cells. To achieve this, the nanosystems, along with free BVZ as a control, were administered directly into the striatum of healthy rats. After 48 h, immunolabeling of brain coronal sections was performed to assess their distribution and cellular internalization in the striatum (Fig. 6).

The images in Fig. 6 showed distinct cellular interactions between the nanosystems and brain cells, with low correlation to in vitro results. PGA-PEG NCs exhibited significant uptake by microglia, suggesting strong recognition and potential degradation of PGA-PEG NCs. However, a low level of colocalization with neurons and no detectable uptake by astrocytes was observed, contrasting with the in vitro data (Fig. 5). This discrepancy could be attributed to the rapid recognition and clearance of PGA-PEG NCs from brain tissue by microglia, thereby limiting their ability to interact with other brain cell types.

Conversely, PGAC14 NAs displayed strong neuronal uptake along with moderate internalization by astrocytes and microglia (Fig. 6A). This pattern indicates a preferential neuronal interaction while avoiding significant phagocytosis by microglia, in contrast to PGA-PEG NCs. This reduced microglial recognition highlights PGAC14 NAs as a promising candidate for neurodegenerative disorders, where neuronal targeting is a key objective [52, 53]. The higher neuronal uptake of PGAC14 NAs compared to PGA-PEG NCs is likely attributed to their surface composition. Previous studies by Silvia Dante and colleagues have reported that negatively charged nanoparticles can preferentially interact with and internalize into neurons due to the electrical activity of neuronal cells, which may create a “charge-driven effect” attracting anionic nanosystems, whereas neutral or positively charged particles exhibit minimal interaction [54].

It should be noted that no colocalization was observed when free BVZ was evaluated (Figure S3), which demonstrates that the internalization of BVZ into brain cells is mediated by its encapsulation within the PGAC14 NAs.

**Fig. 7:**
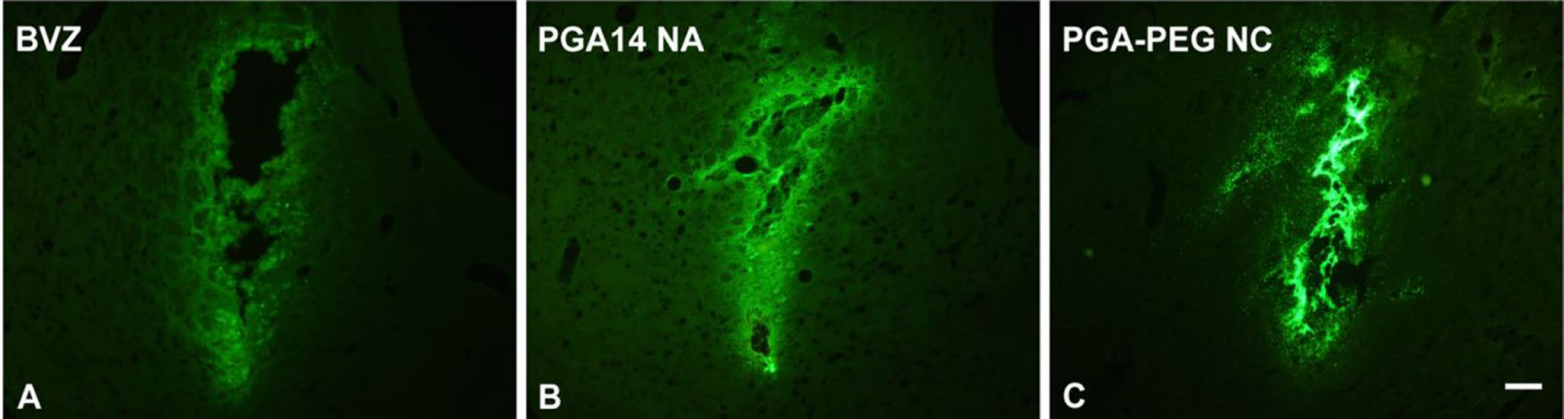
Representative images from coronal striatal sections following administration of free AlexaFluor^488^-labeled BVZ (**A**), and AlexaFluor^488^-labeled BVZ (green) encapsulated in PGAC14 NA (**B**) and PGA-PEG NCs (**C**) nanosystems. Free AlexaFluor^488^-labeled BVZ was used as a control. Coronal brain sections (30 μm thick) were collected at 120 μm intervals. Scale bar: 200 μm

Beyond cellular interaction, a key focus of this study was the diffusion ability of both nanosystems within the brain parenchyma. To assess this, coronal slices were collected to evaluate the volume of distribution for each prototype. As shown in Fig. 7, free BVZ remained largely confined to the injection site after 48 h across all examined sections, whereas both BVZ-loaded nanosystems exhibited improved dispersion compared to free BVZ. However, a distinct difference in distribution profiles was observed between the two prototypes.

**Fig. 8:**
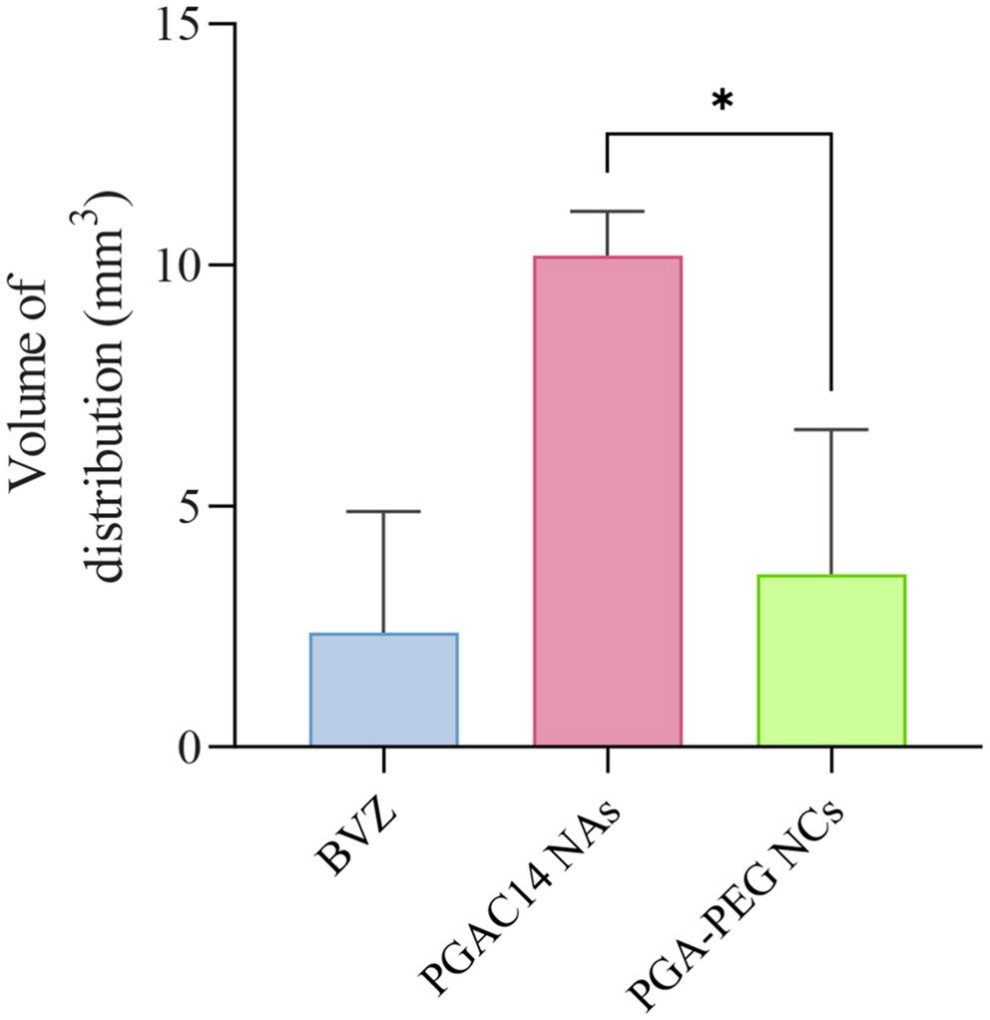
Quantification of the volume of distribution for each nanosystem prototype and free BVZ, calculated based on the total diffusion area and the cumulative depth spanned by the brain sections. Coronal brain sections (30 μm thick) were collected at 120 μm intervals. Data are represented as mean ± SD; *n* ≥ 2. Statistical analysis was performed using an unpaired t-test

As illustrated in Fig. 7, PGA-PEG NCs remained distributed near the infusion site, suggesting their limited diffusion across the brain tissue. However, some fluorescence beyond the injection area was detectable, indicating a moderate distribution volume of approximately 4 mm³ (Fig. 8). These results suggest that while some individual nanosystems may have diffused through the brain parenchyma, the overall distribution remained suboptimal. Given that similar oil-core nanosystems have previously facilitated the diffusion of mRNA-based nanoemulsions up to 3.4 mm from the injection site [8], these findings suggest that PGA-PEG NCs may be subject to rapid microglial recognition and phagocytosis, thereby limiting their diffusion.

In contrast, PGAC14 NAs exhibited significantly enhanced diffusion from the injection site, as shown in Fig. 7. Compared to free BVZ, PGAC14 NAs demonstrated a fourfold increase in distribution volume, reaching 10 mm^3^, confirming widespread dispersion across all examined brain sections (Fig. 8). The superior distribution of PGAC14 NAs compared to PGA-PEG NCs can be attributed to their distinct physicochemical properties. Specifically, PGAC14 NAs possess an ultra-small size of 40 nm, compared to the 80 nm of PGA-PEG NCs. This difference is likely a key factor, as smaller nanoparticles have been reported to diffuse more efficiently through the brain’s interstitial space, which ranges from 38 to 64 nm in a normal brain [50, 55]. Given that the extracellular matrix (ECM) can act as a physical barrier to larger particles, the reduced size of PGAC14 NAs may facilitate their movement through the brain tissue. However, the correlation between particle size and diffusion remains a subject of debate, as nanoparticles larger than 100 nm have also demonstrated robust diffusion following intracranial administration [56].

Therefore, in addition to size, the more negative surface charge of PGAC14 NAs (-10 mV) likely contributed to their enhanced mobility within the brain parenchyma. The brain ECM contains negatively charged components such as glycosaminoglycans and proteoglycans, which can strongly interact with positively charged nanoparticles, leading to their sequestration and limited diffusion [57]. In contrast, the negative zeta potential of PGAC14 NAs likely reduced these electrostatic interactions, allowing for greater freedom of movement through the brain tissue [25]. Furthermore, the lower sequestration of PGAC14 NAs by microglia, as evidenced by the lack of colocalization in Fig. 6A, preventing excessive clearance and further promoting nanoparticle distribution.

Notably, high concentrations of small nanoparticles have been shown to improve brain diffusion by saturating available binding sites within the extracellular matrix, allowing nanoparticles to migrate further from the infusion site [24, 58]. Given the low toxicity observed in vitro and the high BVZ loading capacity of PGAC14 NAs, future studies could explore higher BVZ concentrations to further enhance brain parenchyma diffusion.

**Fig. 9:**
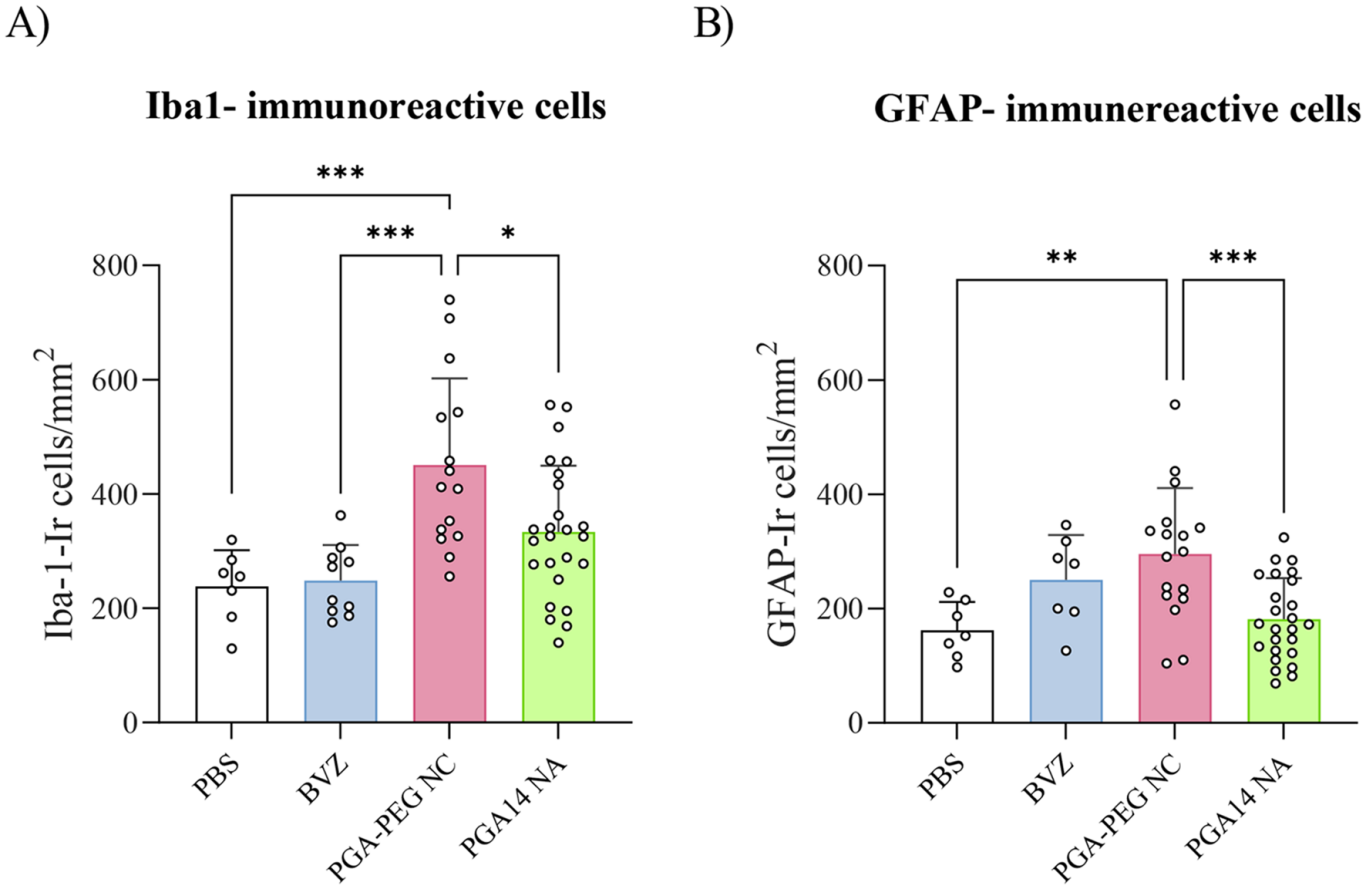
Quantification of microglial and astrocytic responses in peri-injection regions. Density of Iba1-immunoreactive cells (**A**) and GFAP-immunoreactive cells (**B**), expressed as the number of positive cells per square millimeter (cells/mm²), in animals injected with PBS, BVZ, PGA–PEG nanosystems, or PGA14–NA nanosystems. Quantification was performed on immunofluorescence labelled sections using semi-automated analysis with ImageJ software. Data are represented as mean ± SEM; *n* ≥ 7. Statistical analysis was performed using one-way ANOVA *p < 0.05 vs. C; #*p* < 0.05 vs. BVZ; &*p* < 0.05 vs. PGA–PEG NC

Additionally, the impact of local inflammation on brain diffusion was assessed by evaluating glial activation in the area surrounding the intracranial injection. As shown in Fig. 9, both nanosystems induced distinct glial responses in peri-injection regions. PGA–PEG NCs treatment significantly increased both Iba1- and GFAP-immunoreactive cell densities compared to PBS and iba-1 immunoreactive cell density compared to BVZ. In contrast, PGA14 NAs elicited a significantly lower glial response, with reduced numbers of Iba1- and GFAP-positive cells compared to PGA–PEG NCs. These findings suggest a more favorable neuroinflammatory profile for the PGA14 NAs.

Evaluating inflammation is critical to fully understand and interpret the diffusion behavior of intraparenchymal administered nanosystems. In particular, activation of astrocytes and microglia may be accompanied by edema formation and ECM remodeling, which can significantly alter ECM permeability [59, 60]. However, the increased glial activation observed following PGA-PEG NCs administration was accompanied by evident microglial uptake of the nanosystems (Fig. 6B). These results suggest an alternative scenario in which PGA-PEG NCs could have been sequestered by reactive glial cells, thereby limiting their capacity to diffuse further from the injection site. In contrast, the more moderate glial response and absence of microglial internalization of PGA14 NAs suggest a more accurate and representative distribution profile.

Together, these properties highlight PGAC14 NAs as a promising platform for enhancing the diffusion of therapeutic macromolecules in the brain.

## Conclusions

In this study, we successfully developed and compared two different nanosystems, PGA-PEG NCs and PGAC14 NAs, to evaluate their ability to diffuse through the brain parenchyma and intracellular mAb delivery. Our findings showed that the physicochemical properties of these nanosystems play a crucial role in determining both their diffusion within the brain and their interaction with brain cells. PGA-PEG NCs exhibited limited neuronal and astrocytic uptake while showing a high degree of clearance by microglia, which we hypothesized to be a key factor in their restricted brain distribution.

In contrast, PGAC14 NAs exhibited a high neuronal uptake with an enhanced brain diffusion and favorable neuroinflammatory profile. The diffusion was likely driven by their ultra-small size, which enabled them to remain in the extracellular space and penetrate deeper into brain tissue. These results emphasize the importance of nanosystem architecture in optimizing both brain diffusion and cellular targeting.

Overall, PGAC14 NAs emerge as a promising platform for neuronal-targeted delivery and enhanced mAb distribution within the brain. Future studies should investigate how these structural differences impact therapeutic efficacy and explore further optimization of nanocarriers to enhance mAb-based treatments for neurological disorders.

## Supplementary Information

Below is the link to the electronic supplementary material.

Supplementary Material 1
