# Supplementary material for: Delivery of monoclonal antibodies to the brain: the impact of nanocarrier structure
Source: Drug Deliv Transl Res. 2025 Sep 2;16(3):780–96. doi: 10.1007/s13346-025-01957-y (PMC12876119; doi:10.1007/s13346-025-01957-y)
Supplement: Supplementary file 1 — Supplementary Material 1 [file 13346_2025_1957_MOESM1_ESM.docx]

**Table 1. P**hysicochemical properties of both prototypes, PGA-PEG NCs and PGAC14 NAs, with and without AlexaFluor^488^-BVZ

| **Prototype** | **Physicochemical properties** | | | **AE (%)** | **LC (%)** |
| --- | --- | --- | --- | --- | --- |
|  | **Particle size (nm)** | **PDI** | **ζ potential (mV)** |  |  |
| **PGA-PEG NCs (BVZ)** | 78 ± 3 | 0.06 | -1 ± 1 | 99 ± 14 | 4.0 |
| **PGA-PEG NCs (AlexaFluor^488^-BVZ)** | 81 ± 13 | 0.10 | -1 ± 1 | 112 ± 1 | 5 |
| **PGAC14-based NAs (BVZ)** | 44 ± 4 | 0.24 | -10 ± 2 | 64 ± 18 | 21 |
| **PGAC14-based NAs (AlexaFluor^488^-BVZ)** | 43 ± 5 | 0.27 | -2 ± 1 | 69 ± 14 | 22 |

**Figure S1. Dot blot analysis of BVZ-loaded nanosystems showing specificity for VEGF antigen.** A) BVZ-loaded PGA14 NAs B) BVZ-loaded PGA-PEG NCs. Membranes were blotted with recombinant human VEGF (0.05 µg), followed by incubation with free BVZ, BVZ-loaded PGA14 NAs or BVZ-loaded PGA-PEG NCs (BVZ concentration of 0.01 mg/ml). Parallel control conditions included membranes without VEGF antigen to assess nonspecific binding (Controls 1 and 2), and direct blotting of free BVZ onto the membrane as a positive control (Control 3). Blank PGA14 NAs and PGA-PEG NCs were also incubated with VEGF to evaluate potential background signal from the carriers.

**Figure S2. Radiochemical characterization of ^89^Zr-BVZ, ^89^Zr-BVZ-loaded PGA-PEG and ^89^Zr -BVZ-loaded PGAC14 NAs.** A) Radiochemical yield; B) Radiochemical purity; C) Radiochemical stability after 0, 4, 24 and 48 hours of incubation in PBS at 37ºC. BVZ-loaded PGA-PEG NCs without DFO were used as control (Control PGA-PEG NCs). Data are represented as mean ± SD, n≥2. Statistical analysis for comparison between free ^89^Zr -BVZ and ^89^Zr -BVZ-loaded PGA-PEG was done using a Mann-Whitney test (A) and unpaired t-test (B). Multiple unpaired t-test was used for comparison between groups (C).


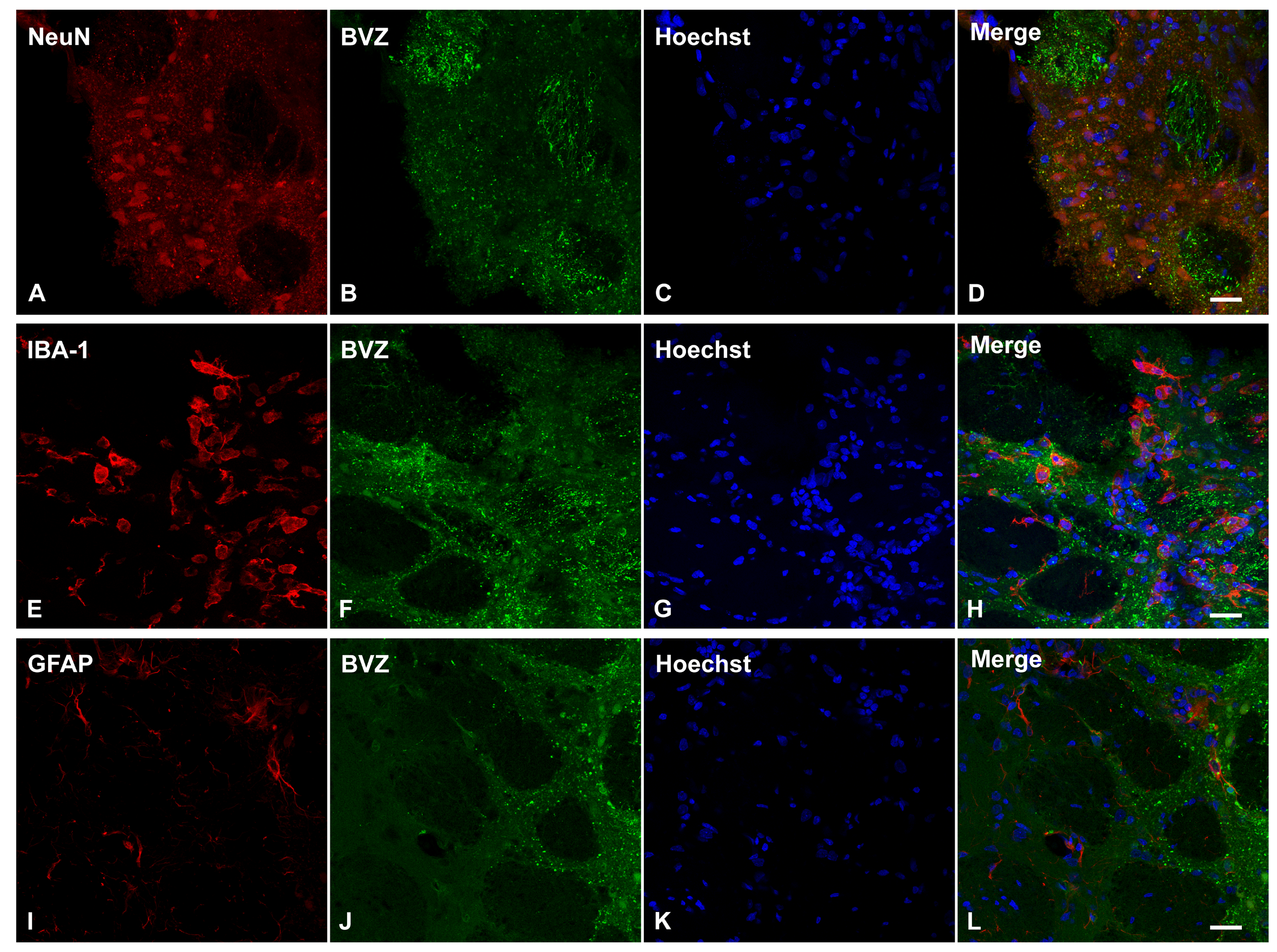


**Figure S3.** Representative confocal micrographs illustrating the lack of internalization of free BVZ in neuronal and glial populations within rat brain tissue. Double immunofluorescence labeling highlights cell-specific markers in red (NeuN for neurons [A]; IBA-1 for microglia [E]; and GFAP for astrocytes [I]), nuclear counterstaining with Hoechst in blue ([C, G, K]), and Alexa Fluor 568-labeled BVZ in green ([B, F, J]). Merged images ([D, H, L]) reveal no detectable colocalization of free BVZ with these cell populations. Scale bar, 20 µm.
